# Analysis of Cutaneous Adverse Drug Reactions (ADR) Reported at an ADR Monitoring Center of a Tertiary Care Teaching Institute in Central India

**DOI:** 10.7759/cureus.53706

**Published:** 2024-02-06

**Authors:** Chaitali A Chindhalore, Ashish V Gupta, Ganesh N Dakhale, Ankita Srivastava

**Affiliations:** 1 Pharmacology, All India Institute of Medical Sciences, Nagpur, Nagpur, IND; 2 Pharmacology, Nandkumar Singh Chauhan Government Medical College, Khandwa, Khandawa, IND; 3 Dermatology, All India Institute of Medical Sciences, Nagpur, Nagpur, IND

**Keywords:** pharmacovigilance, naranjo scale, who-umc scale, urticaria, fixed drug eruption, nsaids, antimicrobials, maculopapular rash, cutaneous adrs

## Abstract

Background

Cutaneous adverse drug reactions (ADRs) are among the most frequent ADRs. Knowledge of the pattern of cutaneous ADRs (CADRs) and causal drugs helps prevent and reduce the incidence of CADR, which in turn reduces the incidence of hospitalization and expenses for the patient.

Objectives

To analyze CADR according to demographic profile, morphological pattern, causative drugs, severity, and outcome in patients suffering from CADRs.

Materials and methods

Retrospective data analysis was conducted in the Adverse Drug Reaction Monitoring Centre (AMC) of the tertiary care teaching institute between February 2020 and September 2023 under the Pharmacovigilance Program of India (PvPI). All ADRs reported were analyzed based on the following parameters: total number of ADRs reported, number of CADRs, information related to demographic parameters, the clinical presentation of CADRs, and suspected medication. Causality assessment was done using the World Health Organisation-Uppsala Monitoring Centre (WHO-UMC) scale. Severity was assessed using a modified Hartwig and Seigel scale.

Results

A total of 125 CADRs were analyzed. Considering the gender-wise distribution, 65 females and 60 males suffered from CADR. The most common drug category responsible for CADRs was antimicrobials (63.2%), followed by topical agents (12.8%). Maculopapular rash (33.6%) was the most common presenting symptom, followed by itching (27.2%). Few patients suffered from serious CADRs such as Stevens-Johnson syndrome (SJS) and toxic epidermal necrolysis (TEN).

Conclusion

A wide clinical spectrum of CADRs ranging from maculopapular rash to fixed-drug eruption to serious SJS was observed in our study. The most common causative agents for CADRs were antimicrobials, followed by topical agents and NSAIDs. For early diagnosis and management of CADRs, it is critical to have data on the potential cutaneous adverse effects of commonly used drugs, to educate the patients regarding common early symptoms of drug reactions (e.g., erythematous rash, edema, urticaria, mucosal erosions, itching, burning of skin, etc.), and to monitor the patient, especially during the start of therapy. To ease the burden of CADRs, a therapeutic plan of anticipating, avoiding, recognizing, and responding to ADRs should be implemented.

## Introduction

The World Health Organization (WHO) defined an adverse drug reaction (ADR) as "any response to a drug which is noxious and unintended and which occurs at doses normally used in man for the prophylaxis, diagnosis, or therapy of disease, or the modification of physiological function" [1]. According to Jha et al., cutaneous symptoms occur in 10-30% of ADRs, with 2-3% being seen in hospitalized patients [2]. A cutaneous ADR (CADR) is an undesirable change in the structure or function of the skin, its appendages, or mucous membranes. It encompasses all adverse events related to drug eruptions, regardless of the etiology. These reactions range from mild to life-threatening, severe CADRs [3].

The incidence of CADRs is one to three percent in developed countries, while the incidence in developing countries is supposed to be higher between 2% and 5%, as reported by Nandha et al. [4].

CADRs with milder signs include maculopapular rash, fixed-drug eruption (FDE), purpura, skin hypopigmentation, itching, urticaria, and various additional manifestations such as bulla and vasculitis. Severe cutaneous adverse responses (SCAR) are CADRs that are potentially fatal. Stevens-Johnson syndrome (SJS), toxic epidermal necrolysis (TEN), acute generalized exanthematous pustulosis (AGEP), and hypersensitivity syndrome or drug reaction with eosinophilia and systemic symptoms (DRESS) are examples of these conditions [5].

Various studies in the past have analyzed CADR based on different parameters. According to a study by Modi et al. [6], out of 2171 ADR, 538 were CADR. Maculopapular rash and itching were the most common presenting symptoms.

Although many studies have been done on the analysis of CADR with varied observations in India, data are limited from central India. Knowledge of the pattern of CADR, causal drugs, and severity helps in the early identification and better management of CADR. The present study was planned with the thought in mind that the study findings will help prevent and reduce the incidence of CADRs, which in turn will reduce the incidence of hospitalization and expenses for the patient in the future. Hence, the present study was planned to analyze CADR reported to the ADR monitoring center according to demographic profile, morphological pattern, causative drugs, causality, and severity.

## Materials and methods

A retrospective analytical study was conducted after obtaining approval from the institutional ethics committee (AIIMS, Nagpur) (IEC/Pharmac/2023/472) and research cell. Retrospective data analysis was conducted in the Adverse Drug Reaction Monitoring Centre (AMC) established under the Pharmacovigilance Programme of India (PvPI) at a tertiary care teaching institute in central India. As a part of the standard protocol of AMC, treating physicians report ADRs to AMC by filling out a spontaneous ADR reporting form, which includes details of each ADR. Each ADR report is then submitted to the National Coordinating Centre (NCC) via “VigiFlow.” All CADRs reported between January 2020 and December 2023 were identified from this database and analyzed.

All ADRs were analyzed based on the following parameters: total number of ADRs reported, number of CADRs, information related to the demographic profile of patients suffering from CADR, the clinical presentation of CADRs, suspected medication, causality, and severity of CADR.

Causality assessment was done using the WHO-Uppsala Monitoring Centre (WHO-UMC) scale [7]. It is based on the clinical-pharmacological components of the case history. ADRs are categorized into “certain,” “probable,” “possible,” and “unlikely,” based on the temporal relationship between drug intake and the onset of reaction, underlying pathology, rechallenge, and de-challenge. Unless the evidence in the report is already compelling without reexposure, rechallenge information with a satisfactory outcome is necessary for “certain.” A rechallenge is not necessary for “probable,” on the other hand. The temporal relationship between the intake of medicine and the event must be “plausible” to qualify as “certain.” The temporal relationship should be reasonable for “probable.” The key difference between “probable” and “possible” is that, in the latter situation, there may be another equally plausible explanation for the event. Causality is “unlikely” if no criteria are fulfilled.

Severity was assessed using the modified Hartwig and Seigel scale [8]. The Hartwig Scale categorizes ADRs into seven levels of severity. Levels 1 and 2 are mild; levels 3 and 4 are moderate; and levels 5, 6, and 7 are severe. ADR is labeled as mild if it is self-limiting and requires no treatment. If ADRs require treatment or increase the length of hospital stay by at least one day, then they are considered to have moderate severity. If ADR is life-threatening or causes permanent harm or death, then it is labeled as severe ADR.

Statistical analysis

Data were entered using Microsoft Excel (Microsoft® Corp., Redmond, WA). The quantitative variables such as age and latency period were summarized as mean (SD) or median (IQR) based on the normality of distribution, and categorical data were summarized using frequency and percentage.

## Results

A total of 436 ADRs were reported between January 2020 to December 2023 at the Adverse Drug Reaction Monitoring (AMC) Centre of tertiary care teaching institute in central India. Out of which, 125 (28.66%) were CADRs. Considering gender-wise distribution, 65 females and 60 males reported CADR.

Age-wise distribution of cutaneous ADR

The mean age of patients who reported to have suffered from CADR was 36.17 ± 17.71 years. The most commonly affected age category was 41-60 years (31.2%), followed by 18-30 years (24.8%) (Table 1).

**Table 1:** Age-wise distribution of cutaneous ADR among patients (n=125)

Sr No	Age category (years)	Distribution of ADR, n (%)
1	≤2	5 (4)
2	3-11	7 (5.6)
3	12-17	11 (8.8)
4	18-30	31 (24.8)
5	31-40	20 (16)
6	41-60	39 (31.2)
7	≥61	12 (9.6)

Clinical presentation of CADR

The latency period for the development of the first sign of CADR after starting drug administration ranges from 10 minutes to 15 days, depending on the type of CADR. The median latency period in terms of hours was 72 (5-189).

Cutaneous manifestations of ADRs include maculopapular rash (33.6%), followed by pruritus (27.2%), acneiform eruption (10.4%), urticaria (10.4%), fixed-drug eruption (6.4%), blisters (3.2%), and hypomelanosis (2.4%). Few CADRs manifested as SJS, toxic epidermal necrolysis (TEN), erythema multiforme, and erythroderma (Table 2).

**Table 2:** Clinical presentation of CADR with frequency (n=125)

Reported cutaneous ADR (preferred term)	Frequency, n (%)
Rash maculopapular	42 (33.6)
Pruritus	34 (27.2)
Acneiform eruption	13 (10.4)
Urticaria	13 (10.4)
Fixed drug eruption	8 (6.4)
Blisters	4 (3.2)
Hypomelanosis	3 (2.4)
TEN	3 (2.4)
Erythema multiforme	3 (2.4)
Erythroderma	1 (0.8)
SJS	1 (0.8)

Drug categories responsible for CADR

The most common causal drug category was antimicrobials 79 (63.2%), followed by topical agents (skin ointment containing a combination of antifungal, corticosteroid, and antibacterial agents; 16, 12.8%), NSAIDs (8, 6.4%), and steroids (7, 5.6%) (Table 3).

**Table 3:** Drug categories responsible for CADR *Topical agents include skin ointment containing a fixed-dose combination (FDC) of different antifungal, corticosteroid, and antibacterial agents.

Drug categories responsible for CADR	Frequency (n (%))
Antimicrobial agents	79 (63.2)
Topical agents*	16 (12.8)
NSAIDs	8 (6.4)
Corticosteroids	7 (5.6)
Immunosuppressants	5 (4)
Antiepileptics	5 (4)
Hematinics	5 (4)

Among antimicrobials, beta-lactam antibiotics were responsible for 29 (36.7 %) reported cutaneous ADRs (Table 4).

**Table 4:** Antimicrobial drug classes responsible for CADR *Number indicates the frequency of reported CADRs that were caused by respective antimicrobial agents. Numbers in parentheses indicate percentages.

Antimicrobial agents	Frequency of CADR (number (%))*
Antibacterial agents	
Beta lactam antibiotics	29 (36.7)
Macrolides	14 (17.72)
Fluoroquinolones	12 (15.18)
Anti-TB agents	7 (8.86)
Anti-amoebic agents	4 (5.06)
Sulfonamides	4 (5.06)
Antifungal agents	
Amphotericin B	4 (5.06)
Azole derivatives	1 (1.26)
Antiviral agents	
Favipiravir	3 (3.37)
Remdesivir	1 (1.26)

Causality and severity assessment of CADR

Causality assessment of CADRs according to the WHO-UMC scale revealed that 4.8% of CADRs belonged to the category “probable,” while 95.2 % of CADRs belonged to the category “possible.”

The severity of reported CADRs was assessed using a modified Hartwig and Seigel scale. Seventy-two (57.6%) CADRs were mild, 38 (30.4%) were moderate, and 15 (12%) were severe. Severe CADRs that were reported to the ADR monitoring center are as follows: TEN (3), SJS (1), erythroderma (1), erythema multiforme (3), blisters (2), urticaria (3), and extensive rash maculopapular (2).

## Discussion

The most common presentation of ADRs is cutaneous responses. A wide variety of medications may be responsible for the development of CADR. The cutaneous signs range from a simple maculopapular rash to the potentially fatal SJS/TEN. A drug can cause several morphological forms of reactions, and simultaneously, various drug classes can cause the same drug reaction pattern. Some severe CADRs can cause significant morbidity and even death, as reported in previous studies [9,10]. The development of CADRs is a common cause of treatment noncompliance. Failure to warn a patient about potential adverse effects, prescribing a drug to a patient with a known allergy, or prescribing medication with cross-reactivity to a previously sensitized patient are all typical medicolegal issues that must be handled. Although cutaneous reactions are prevalent, data on their prevalence, severity, and outcome is limited.

In the present study, a total of 125 CADRs were reported. CADRs predominance was seen in female patients as compared to male patients, which is in concordance with the study conducted by Mahatme et al. [9]. In contrast, a study by Modi et al. [6] reported male preponderance. Differences in healthcare-seeking approaches in various regions may explain this fact. In the present study, CADRs were mostly found in the age group of 41-60 years. In contrast, most patients experiencing CADRs were in the age group of 21-39 years as reported by Modi et al. [6].

Among various cutaneous manifestations of drug reactions, the maculopapular rash was most frequently documented (33.6%), in conformity with the study done by Nandha et al. [4]. Fixed-drug eruption was the most commonly reported CADR, as reported by Sharma et al. [11], whereas urticaria was the commonest manifestation of CADR, as reported by Mahatme et al. [9].

In the present study, we also saw some fatal CADRs in the form of TEN (2.4%) and SJS (0.8%). However, the incidence of life-threatening CADRs such as SJS and TEN was found to be higher in a nine-year study from south India by Sushma et al. [10]. The Italian study concluded that the incidence of TEN and SJS was 0.2% and 1.82%, respectively [12]. Underreporting may be one of the reasons for the low incidence of fatal CADR.

In the current study, antimicrobials were the most common causative drugs (63.2%), which is like the study findings stated by Nandha et al. [4] and Choon et al. [13]. An analysis of 10-year data from Singapore by Wong et al. [14] stated that systemic antibacterial drugs were most implicated (43.5%), followed by anti-inflammatory and antirheumatic products (16.2%) and analgesics (9.0%). On the contrary, a study by Murthy et al. [15] concluded that anticonvulsants (24.7%) were the most common cause of CADR, followed by NSAIDs (22.5%), antibiotics (20.9%), followed by antiretrovirals (ART; 12.7%) and antituberculous drugs (ATT; 6.7%). This variation could be because of variations in drug utilization patterns. Other reasons may include variations in prevalent disease conditions in different areas. The prevalence of ADR might be high if the drug is commonly prescribed despite of unfavorable adverse effect profile.

In the present study, topical agents containing an FDC of antibacterial and antifungal drugs with corticosteroids were responsible for 12.8% CADR. Although many of these FDCs are irrational, these FDCs were rampantly prescribed by general practitioners or self-administered by patients in the pursuit of producing temporary and rapid relief. This has resulted in a lot of CADR-like skin atrophy, telangiectasia, purpura, acneiform eruptions, and hypopigmentation. 

Among antimicrobials, beta-lactam antibiotics were causative agents in most patients as reported in previous studies [13]. This could be ascribed to the widespread use of beta-lactam antibiotics in our setup or to regional differences in the usage of antimicrobial agents.

In our study, the majority of CADRs were possibly related to causal drugs. During causality assessment, most of the time ADR belongs to the category “possible” because of the presence of underlying pathology, concomitant medications, and availability of limited information. Previous studies also reported the same [6]. In a study by Ciddhavaduta et al. [16], the majority of CADR (92.5%) were probably caused by suspected medication, which is against the present study findings. In the present study, we determined causality based on the WHO-UMC scale only, which is a limitation of the present study.

The majority of observed ADR belongs to the mild category as per the modified Hartwig and Seigel scale for severity. In contrast, a study by Ciddhavaduta et al. [16] stated that most of the CADRs belonged to moderate category under severity assessment.

## Conclusions

A wide clinical spectrum of CADRs ranging from maculopapular rash to FDE to life-threatening SJS was observed in our study. The most common causative agents for CADRs were antimicrobials, followed by topical agents and NSAIDs. For early diagnosis and management of CADRs, having data on the potential cutaneous adverse effects of commonly used drugs is critical. CADRs often involve allergic reactions to drugs. Identifying these reactions early is crucial for discontinuing the offending drug and preventing further exposure.
